# Evaluation of microneedling with and without injectable-platelet rich fibrin for gingival augmentation in thin gingival phenotype-A randomized clinical trial

**DOI:** 10.1016/j.jobcr.2023.10.008

**Published:** 2023-12-15

**Authors:** Chetana, Sangamithra Sidharthan, Gopalakrishnan Dharmarajan, Shraddha Iyer, Mariam Poulose, Meghana Guruprasad, Dhakshay Chordia

**Affiliations:** a Department of Periodontology, Dr. D.Y. Patil Dental College and Hospital, Dr. D.Y. Patil Vidyapeeth, Sant Tukaram Nagar, Pimpri, Pune, 411 018, Maharashtra, India; b Department of General Surgery Saveetha Medical college, Thandalam, Chennai 602105, Tamil Nadu, India

**Keywords:** Gingiva, Soft tissue augmentation, Microneedling, Plasma platelet rich fibrin

## Abstract

**Objective:**

The purpose of the current study was to investigate the effect of micro needling (MN) on gingival thickness (GT) and keratinized tissue width (KTW) in individuals with thin gingival phenotypes, either with or without injectable platelet-rich fibrin (i-PRF).

**Materials and methods:**

In this randomized, split-mouth clinical trial, 15 systemically healthy patients, with thin gingival phenotype (<1.5 mm) were randomly treated with MN + i-PRF and MN. MN was performed on one side, and MN + i-PRF on the contralateral side of the same arch at 4 sessions with 10-day intervals. GT; KTW the primary outcome and Plaque index; gingival index Secondary outcome were assessed at baseline and at 1st, 3rd, and 6th months post-treatment.

**Results:**

The results of the present study showed that both techniques demonstrated a statistically significant increase in GT. GT showed a statistically significant increase from baseline (0.453 ± 0.069 mm in MN, 0.451 ± 0.069 mm in MN + i-PRF) (p = 0.81) to 1 month (0.567 ± 0.075 mm in MN, 0.649 ± 0.075 mm in MN + i-PRF) (p < 0.001*). A follow-up at 3rd month (0.566 ± 0.076 mm in MN, 0.647 ± 0.091 mm in MN + i-PRF) (p < 0.001*) and 6th month (0.564 ± 0.076 mm in MN, 0.644 ± 0.089 mm in MN + i-PRF) (p < 0.001*) showed a statistically significant increase. Intergroup comparison showed a statistically significant GT increase in MN + i-PRF sites at all the time intervals. No, statistically significant difference in KTW was observed in both groups from baseline to 6 months.

**Conclusions:**

The utilization of MN + i-PRF stands as a minimally invasive, non-surgical method to improve GT. Interestingly, using i-PRF as an additional component demonstrated more favorable outcomes compared to using MN alone in enhancing tissue thickness.

## Graphical abstract

**Figure:**
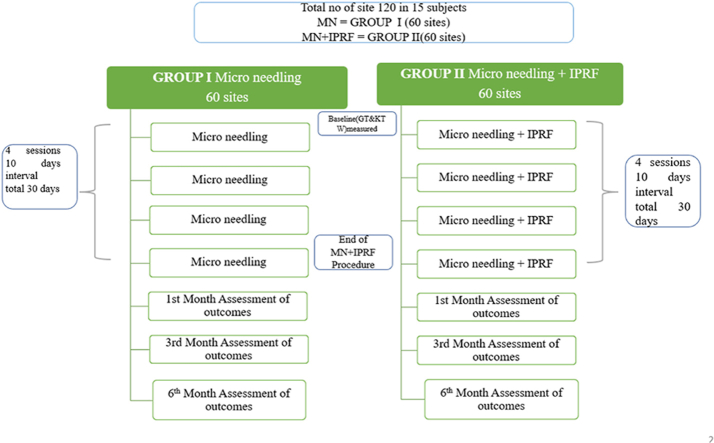

## 1 Introduction

Biotype (Genetics) refers to a group of organisms having the same specific genotype while phenotype refers to the appearance of an organism based on combination of multiple factors like environment and clinical intervention that may be specific to site (including the expression of biotype). Thus, phenotype indicates the dimensional change with respect to time and various other factors, hence can be modified while genotype cannot be.^1^

It is to be understood that, gingival phenotype (three-dimensional gingival volume) that is inclusive of gingival thickness (GT), keratinized tissue width (KTW) when considered along with buccal bone plate thickness (bone morphotype), is referred as “periodontal phenotype”. However, in this study GT and KTW are considered, hence we have used the term “gingival phenotype”.^1^ Also, depending on the size, texture, location, and shape of the gingiva and alveolar process, each person has a distinct “Gingival Phenotype” that affects their periodontal response to inflammation and course of therapy.^2^

The characteristics of a thin gingival phenotype include a narrow triangular-shaped crown, a soft convexity along the cervical aspect, proximal contacts at the incisal one-third of the tooth, inadequate keratinized tissue, translucent and thin friable gingiva with thickness less than 1.5 mm and thin alveolar bone, while those of a thick phenotype include a square and pronounced cervical bulge, contact point that are located more apical with a broad keratinized gingiva and thickness of 2 mm.^3^^,^^4^ Individuals with thin-phenotype are more likely to experience gingival recession in the context of trauma and inflammation,^4^ however the incidence of recession is more common after a successful periodontal therapy of a periodontal pocket^5^

The best evidence consensus (BEC) established a model in 2016 with the goal of defining factors to maintain periodontal and peri-implant health and concluded that phenotype modification therapy (PhMT) is an aid for the maintenance or improvement of dental health, especially before undergoing major restorative and orthodontic therapy. The BEC highlights the features of thick and thin gingiva/peri-implant phenotypes, and it was evident that the thin phenotype increases the risk for any form of recession and inflammation.^6^^,^^7^

PhMT intervention is performed with the help of free gingival grafts, connective tissue grafts, dermal grafts (acellular), and enamel matrix derivatives for increasing soft tissue thickness that enhances restorative, orthodontic and therapeutic outcomes.^8^ Among these, subepithelial connective tissue grafts prove to be the “gold standard” for augmenting the gingiva. However, these procedures demand surgical dexterity and exhibit a low rate of patient compliance.^9^

Percutaneous collagen induction therapy, now termed as microneedling (MN), is a novel dermatological therapeutic modality that is minimally invasive.^10^ MN causes micro-injuries, resulting in minor superficial bleeding, and a cascade of wound-healing factors are produced comprising various growth factors that attract neoangiogenesis, and hence neocollagenogenesis.^11^

The growth factors from stem-cell conditioned media and platelet concentrates for percutantaneous collagen process is utilized with microneedling to improve the overall effect of therapy.^12^ For more than a decade, platelet-rich fibrin (PRF) has been utilized as a biocompatible regenerative material in various dental procedures, such as the management of intrabony defects, gingival recession, furcation defects, and preservation of the extraction socket.^13^ Choukroun et al. conducted an in vitro investigation that revealed enhanced neovascularization and wound healing with faster tissue remodelling in the absence of infectious events with i-PRF.^13^ In 2014, injectable platelet-rich fibrin (i-PRF) was developed with a lower centrifugation speed for blood in non-glass centrifugation tubes resulting in liquid platelet-rich fibrin.^14^^,^^15^

The synergistic effects of MN and i-PRF on neo angiogenesis, neocollagenogenesis, and wound healing have been considered in this study and aims to evaluate the effects of standalone MN and MN in combination with i-PRF on the GT and KTW in individuals with thin gingival phenotypes.

## 2 Materials and methods

**Fig. 1:**
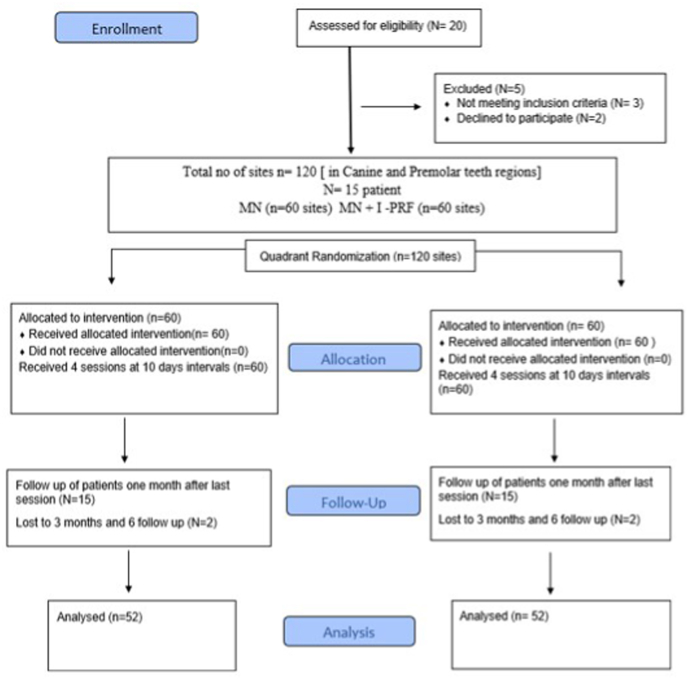
A CONSORT flow chart of the study.

Patients from the Department of Periodontology at Dr.D.Y.Patil Dental College and Hospital in Pimpri, Pune, comprise the study population. The study protocol was created in compliance with the 1975 Helsinki Declaration, as amended in 2013, and was approved by the university's scientific and ethics council (DPU/484/4/2021). This is a split-mouth randomized, triple-blinded clinical study reported in accordance with the CONSORT 2010 guidelines (Fig. 1).^16^ Based on available literature,^17^ the sample size was calculated using G* Power 3.1.9.2 software and was determined to be 15 participants with 120 sites (60 sites for each group). The level of significance (α) was set at 0.05, effect size at 0.80 to achieve a power of 80 %.

### 2.1 Inclusion criteria

Patients in the age group of 18–55 years from both genders without any blood disorder and willing to undergo treatment with informed consent were included. Sites with thin gingival phenotype (gingival thickness<1.5 mm) in maxillary and mandibular canine and 1st premolars region,^4^ Plaque index (PI) < 1,^18^ Gingival index (GI) < 1,^19^ bleeding on probing score of <10 %^20^ were considered.

### 2.2 Exclusion criteria

Patients undergoing active orthodontic treatment, taking any medication that may cause gingival enlargement or blood thinners, pre-existing mucogingival deformities, bruxism, any previous history of periodontitis or periodontal therapy, pregnant/lactating women, use of tobacco in any form, and any systemic diseases.

A detailed clinical examination, case history, and written informed consent were obtained from all the patients along with a complete hemogram. Instructions regarding proper oral hygiene measures was given. A full-mouth phase I therapy was performed. A periodontal re-evaluation was done after 4–6 weeks of phase I therapy and patients exhibiting a gingival bleeding index of less than 10 % were recruited in the study.

The following clinical parameters was recorded at baseline,1st, 3^rd^and 6th months postoperatively. Gingival thickness, Keratinized tissue width

**Fig. 2:**
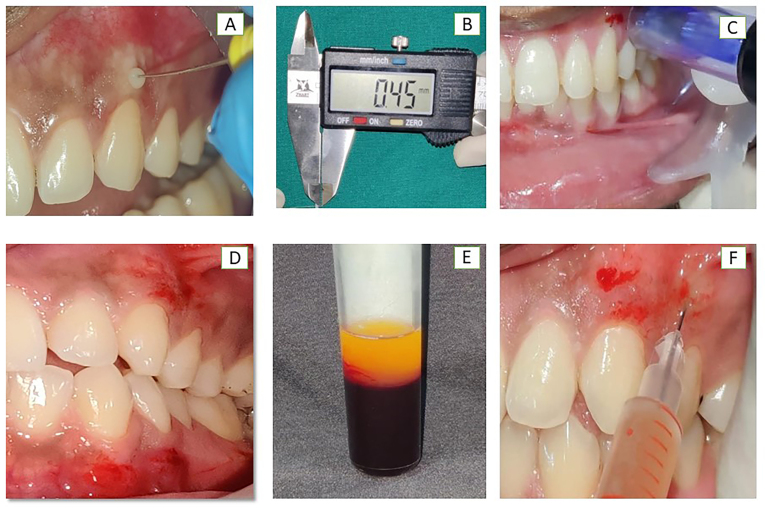
**A) B)** Representative image for measuring GT by transgingival probing for both groups **C) D)** Uniform pin-point bleeding after microneedling (Dr. Pen)™ in both the groups; **E)**Procuring i-PRF; **F)** injecting i-PRF in Group 2.

**Measurement of Gingival Thickness:** The gingival phenotype was categorized as either thin or thick according to the visibility of the underlying periodontal probe through sulcus probing of the mid-facial aspect of the tooth was performed.^5^ The gingiva was anesthetized by a topical gel. The gingival thickness was measured at 1.5 mm apical to the gingival margin in the mid-buccal region of the selected tooth with an already established protocol.^9^^,^^17^ (Fig. 2A and B).

**Keratinized Tissue Width (KTW)**: The distance from the free gingival margin to the MGJ was measured with periodontal probe at the mid buccal region.^17^

**Mucogingival Junction (MGJ):** The borderline between movable and immovable tissue, where tissue mobility is assessed by running a periodontal probe horizontally from the vestibule towards the gingival margin with light pressure, is utilized for MGJ assessment.^21^

### 2.3 Randomization, allocation, and blinding

Allocation of specific treatment to the procedure site was priorly decided by computer-generated random sequence. 60 sites were assigned to group A and 60 sites to group B.

In this clinical trial; participants, outcome assessor (S.S) and statistician (S.K) were blinded to the type of intervention being allocated.

The povidone-iodine solution was used to ensure extraoral asepsis and intraoral asepsis was ensured with a pre-procedural rinse of 0.2 % chlorhexidine gluconate. In both groups, Micro-needling was done using a derma pen (Dr. Pen Ultima A6) ™ at the selected site after application of an anesthetic gel. The Derma Pen was vertically inserted into the tissue until it hits the bone, and the procedure was carried out on keratinized gingiva.^17^ The treatment endpoint was identified as uniform pin-point bleeding, which was easily controlled. In group 2 venous blood sample was collected from each patient using a 20-ml injector, separated into two i-PRF tubes of 10 ml each containing no anticoagulant, and centrifuged at room temperature for 3 min at 700 rpm (60 g force) with the Choukroun PRF Duo Centrifuge.^15^ The i-PRF obtained was injected at the selected site into the keratinized tissue using a 0.25 mm (31G) x 6 mm needle, (BD GlideTM needle) insulin syringe until the blanching and fullness of the gingiva were noted.^17^ A total of 4 sessions were performed on the individuals at 10-day intervals.^14^^,^^17^ (Fig. 2C and D,E,F) The clinical measurements of individuals were taken at baseline, 1st, 3rd^,^ and 6th month during the follow-up sessions after the procedure. Post-procedural instructions were given with a reinforcement of oral hygiene maintenance. Analgesics (Paracetamol 500 mg S–O–S) along with Chlorhexidine 0.2 % rinse twice daily for 14 days was prescribed. Post-operative measurements were taken for Group 1 and 2 (Fig. 2A and B).

### 2.4 Statistical analysis

Statistical Package for Social Sciences [SPSS] for Windows Version 22.0 Released 2013. Armonk, NY: IBM Corp., was used to perform all the statistical analyses. Descriptive analysis of all the explanatory and outcome parameters was done using frequency and proportions for categorical variables, whereas in mean and standard deviation for continuous variables. Chi square test was used to compare distribution of teeth involved between two groups. Independent student “t” test was used to compare the mean gingival thickness and keratinized tissue width between two groups at different time intervals. Friedman's Test followed by wilcoxon signed rank post hoc test was used to compare the mean plaque & gingival Index scores between different time intervals among study patients. Repeated Measures of ANOVA followed by Bonferroni's post hoc Test was used to compare the mean gingival thickness and keratinized tissue width between different time intervals in each group. The level of significance was set at p < 0.05.

## 3 Results

A total of 120 sites were treated in 15 individuals with a mean age of 26 ± 4 years, [60 %] males and [40 %] females (Table 1).

**Table 1:** Teeth distribution and gender demographic of selected sites among study patients (chi square test; p-value <0.001*); plaque index and gingival index scores between different times intervals (Friedman's Test; p-value <0.001*).

Tooth	Group1 (Microneedling)		Group 2 (Microneedling + i-PRF)		p-value
	n	%	n	%	0.99
Tooth 13	8	53.3 %	7	46.7 %	
Tooth 14	8	53.3 %	7	46.7 %	
Tooth 23	7	46.7 %	8	53.3 %	
Tooth 24	7	46.7 %	8	53.3 %	
Tooth 33	7	46.7 %	8	53.3 %	
Tooth 34	7	46.7 %	8	53.3 %	
Tooth 43	8	53.3 %	7	46.7 %	
Tooth 44	8	53.3 %	7	46.7 %	
Variable	Category	n	%		
Gender	Males	9	60.0 %		
	Females	6	40.0 %		

Secondary outcome	Baseline	1 Month		3 Months		6 Months		p-value
	Mean	SD	Mean	SD	Mean	Mean	SD	
PI	0.93	0.24	0.48	0.21	0.53	0.52	0.14	<0.001*
GI	0.93	0.24	0.48	0.21	0.53	0.52	0.14	<0.001*

The loss to follow-up at 3 & 6 Months was n = 2 and it accounts to 13.3 % and it is well within the acceptable rate of attrition in interventional clinical studies [<15 %].^23^ So, no ancillary analysis was needed in the present study to compensate for the loss to follow-up patients.

**Gingival Thickness;** The mean gingival thickness at baseline in Group 1 was [0.454 ± 0.068 mm] and Group 2 was [0.451 ± 0.069 mm] that was statistically insignificant. The mean gingival thickness at 1 Month was significantly higher in Group 2 [0.649 ± 0.075 mm] as compared to Group 1 [0.567 ± 0.075 mm]. The mean gingival thickness at 3 and 6 Months was significantly higher in Group 2 [0.647 ± 0.091 mm] as compared to Group 1 [0.566 ± 0.076 mm] (Table 2) Multiple comparison of mean difference between different time intervals in group 1 and group 2 demonstrated a statistically significant increase in mean gingival thickness at 1st, 3rd and 6th month as compared to baseline. Further no significant difference was demonstrated in the mean gingival thickness between 1st, 3rd and 6th month. In group 2, the mean gingival thickness was significantly increased at 1st, 3rd and 6th month as compared to baseline and the difference observed was statistically significant. (Table 2).

**Table 2:** Comparison of mean values of different clinical parameters between 2 Groups at time intervals (independent student *t*-test; p < 0.001*); multiple comparison within groups for GT (Bonferroni's post hoc test; p-value <0.01*).

Primary outcome	Time	Group 1		Group 2		p-value
		Mean	SD	Mean	SD	
**GT (In mm)**	Baseline	0.454	0.068	0.451	0.069	0.81
	1 Month	0.567	0.075	0.649	0.089	<0.001*
	3 Months	0.566	0.076	0.647	0.091	<0.001*
	6 Months	0.564	0.076	0.644	0.089	<0.001*
**KTW (In mm)**	Baseline	3.50	0.50	3.50	0.50	1.00
	1 Month	3.57	0.56	3.50	0.50	0.50
	3 Months	3.58	0.57	3.50	0.51	0.50
	6 Months	3.50	0.57	3.58	0.51	0.50

Gingival thickness-Multiple comparison within Groups	Time	Time	Mean Diff.	95 % CI for Diff.		p-value
				Lower	Upper	
Group 1	Baseline	1 Month	−0.113	−0.128	−0.099	<0.001*
		3 Months	−0.113	−0.126	−0.100	<0.001*
		6 Months	−0.112	−0.124	−0.099	<0.001*
	1 Month	3 Months	0.000	−0.003	0.003	1.00
		6 Months	0.002	−0.002	0.005	1.00
	3 Months	6 Months	0.001	0.000	0.003	0.20
Group 2	Baseline	1 Month	−0.201	−0.220	−0.182	<0.001*
		3 Months	−0.196	−0.214	−0.177	<0.001*
		6 Months	−0.193	−0.211	−0.175	<0.001*
	1 Month	3 Months	0.005	0.001	0.008	0.003*
		6 Months	0.007	0.001	0.014	0.02*
	3 Months	6 Months	0.003	−0.002	0.007	0.57

**Keratinized Tissue Width;** The mean keratinized tissue width at baseline in Group 1 was [3.50 ± 0.50 mm] and Group 2 was [3.50 ± 0.50 mm] that was no statistically significant. The mean Keratinized Tissue Width at 1st, 3rd & 6th month in Group 1 was [3.58 ± 0.57 mm] and in Group 2 was [3.50 ± 0.51 mm] that was not statistically significant **(**Table 2).

**Plaque Index;** The mean plaque index score at baseline was 0.93 ± 0.24, at 1st month was 0.48 ± 0.21, at 3rd month was 0.53 ± 0.14 and at 6th month was 0.52 ± 0.14. This difference in the mean plaque index Scores between different time intervals was statistically significant.

Multiple comparison of mean difference between different time intervals demonstrated that the mean plaque scores was significantly reduced at 1st, 3rd and 6th month as compared to baseline (Table 1).

**Gingival Index;** The mean gingival index scores at baseline was 0.93 ± 0.24, at 1st Month was 0.48 ± 0.21, at 3rd month was 0.53 ± 0.14 and at 6th month was 0.52 ± 0.14. This difference in the mean gingival index scores between different time intervals was statistically significant.

Multiple comparison of mean difference between different time intervals demonstrated that the mean gingival scores was significantly reduced at 1st, 3rd and 6th month as compared to baseline (Table 1).

However, the assessment of the factors-plaque and gingival index was done to understand the patient's overall oral hygiene status during the study period.

### 3.1 Correlation of GT and KTW

The gingival thickness shows a significant strong correlation with keratinized tissue width in Group 1 & 2 (‘r’ = 0.67 & 0.70) respectively and the finding was statistically significant at baseline. In Group 1 & 2 the 1st month follow up there was a moderate and strong correlation between GT& KTW (‘r’ = 0.47 & 0.71) respectively that was statistically significant.

In both the groups, follow up at 3rd and 6th month showed a moderate correlation (‘r’ = 0.44 & 0.43) and strong correlation (‘r’ = 0.62 & 0.70) respectively between GT & KTW and the findings were statistically significant (Table 3).

**Table 3:** Correlation between gingival thickness & keratinized tissue width at different time intervals in Group 1 & Group 2 (Pearson correlation test; p-value <0.001*).

Group	Parameter	values	Baseline	1 Month	3 Months	6 Months
Group 1	GT & KTW	r	0.67	0.47	0.44	0.43
		p-value	<0.001*	<0.001*	0.001*	0.002*
Group 2	GT & KTW	r	0.70	0.71	0.69	0.70
		p-value	<0.001*	<0.001*	<0.001*	<0.001*

## 4 Discussion

It is understood that phenotype modification therapy can maintain dental health and improve the response to restorative, implant, and orthodontic therapy.^7^ In order to prevent gingival recession before orthodontic tooth movement, soft tissue PhMT improves periodontal health, which provides healthy and stable tissue.^8^ There is vast evidence of various invasive techniques for the increase in soft tissue thickness, however, there is patient discomfort and an increased morbidity rate.^9^ A recent addition to PhMT includes minimally invasive techniques like microneedling^8^^,^^17^ and the use of PRF concentrates substituting more invasive therapeutic regimens with microneedling^17^^,^^23^; that have proven to release cytokines, neo-angiogenesis, and neocollagenesis, which activate regenerative mechanisms.^22^ And also, i-PRF concentrates provides an abundance of angiogenic factors like the vascular endothelial growth factor (VEGF).^22^, ^24^ This study evaluated the outcome of microneedling and i-PRF on GT in subjects exhibiting thin gingival phenotype.

While gingival phenotype can be evaluated in a consistent and reliable manner, periodontal phenotype requires digital imaging techniques. Transgingival probing is claimed to be a simple, standardized, reproducible, affordable, and precise method for measuring GT.^25^ Though Digital Imaging and Communications in Medicine (DICOM) and Standard Tessellation Language (STL) files have shown the highest agreement with histology measurement when compared to bone sounding, the clinical significance is to be researched further.^26^ Hence, transgingival probing was chosen over other methods. In this study, topical anesthesia was opted over infiltration to avoid any bias caused by volumetric changes of injecting the anesthetic solution and simultaneously achieving patient comfort.

The results of the present study suggest that both techniques demonstrated a statistically significant GT increase at 6 months post-treatment. GT showed a statistically significant increase from baseline,to 1 month in both the groups. A follow-up at the third and sixth months revealed stable gingival thickness in group 1 and a statistically significant increase in group 2, respectively. However, a negligible reduction in the thickness observed from 1 month to 6 month in both the groups could be attributed to collagen turnover and functional stresses. And, also Intergroup comparison showed a statistically significant GT increase in MN + i-PRF sites. Studies by Ozsagir et al. 2019,^17^ and Fotani et al. 2019^23^ also substantiate that MN + i-PRF effectively increases GT. Nevertheless, MN group has a significant increase in the GT irrespective of i-PRF. Based on the results it could be cautiously interpreted that, if MN is done without i-PRF, the whole process will be exponentially less invasive and equally effective. This increase in gingival thickness is more likely to prevent further development of new recession defect sites.^27^

A statistically no-significant difference in KTW was observed in both groups from baseline to 6 months. However, this could be ignored as the primary objective was to augment the GT. As this procedure was performed in non-recession sites exhibiting adequate KTW and a limited follow-up of 6 months, we were not able to establish evidence on changes in KTW as observed in literature with other substitutes. GT and KTW can alter the gingival margin level in a significant manner, but are minimally associated and can be relatively independent. As a result, one of these soft tissue parameters could be present or missing at any site, or they may both be present in varying degrees.^8^

According to dermatology literature,^10^, ^11^, ^12^, ^28^ the MN technique generally recommends multiple sessions about three to five sessions scheduled two to four weeks apart to improve acne scars by 70 %. Based on the available evidence on MN, there is a peak in the total collagen production during the wound-healing within one to two weeks^29^; additionally, resorption of PRF occurs in about 2 weeks, and highest peak of release of growth factor from i-PRF is at around 10 days.^13^ It is also to be understood that, the resting membrane potential of cells is −70mV and when needles come near the membrane of the cells, the resting membrane potential increases quickly to −100mV, and thus, multiple sessions trigger repeated increased cellular activity and hence release of various proteins, and growth factors.^29^ It is with all this evidence that a combination of MN and i-PRF was advocated to aid in the augmentation of GT in our study.

In this study, dermapen,a device that uses disposable needles that has a narrow needle tip making it more convenient to treat narrow areas like gingiva. Also, the design overcomes the difference in pressure application and penetration depth. Micro punctures were created using derma pen microneedles which produced a controlled injury and minimal superficial bleeding. and is vastly used in percutaneous collagen induction therapy.^5^ i-PRF was prepared according to Choukroun's protocol. Post administration, the liquid fibrinogen maintains its liquid form for around 15–20 min before it coagulates to form an autologous fibrin binder (AFB).^14^^,^^15^ This injected PRF is used in our study to enhance matrix proliferation and in turn aid in gingival thickness augmentation.^17^^,^^23^

In this study, the procedures were performed at the canine and 1st premolar site in four sessions at a 10-day interval in the contralateral maxillary and mandibular region, as they are vulnerable to gingival recession since they are placed labially^30^ and the KTW width, is minimal in this area. This site selection was also complementary to prevent the effect of i-PRF on the contralateral sites.

The non-evaluation of patient-reported experience measures (PREM) and patient-reported outcomes measures (PROMs) is one of the study's limitations. A follow-up period of a longer term will also be required to determine the stability of the augmented tissue and KTW. More research with histological evaluation and a larger sample size will provide us with more reliable results.

## 5 Conclusion

Gingival recession is more likely in patients with thin gingival tissue and narrow gingival width. This risk is enhanced by orthodontic therapy and may become clinically apparent after a period of time. Considering positive effects of i-PRF with MN than standalone MN procedure on the soft tissue thickness this minimally invasive PhMT in thin phenotypes can aid in long-term gingival tissue stability, periodontal health, associated with limited patient concern prior to further treatment modalities.
